# 1847. Overview of Infective Endocarditis in Patients with Enterococcus Bacteremia from National Standpoint

**DOI:** 10.1093/ofid/ofac492.1476

**Published:** 2022-12-15

**Authors:** Premalkumar M Patel, Garrett Van Ostran, Nicholas Camps

**Affiliations:** Mount Sinai Medical Center of Florida, Hollywood, Florida; Mount Sinai Medical Center, Miami Beach, Florida; Mount Sinai Medical Center, Miami Beach, Florida

## Abstract

**Background:**

Enterococci are the third most common hospital-acquired infection responsible for high morbidity and mortality, and are associated with high-grade vascular infections like infective endocarditis (IE). Our objective of the study is to discuss the outcomes of IE among patients with Enterococcus Bacteremia (EB) using ICD-10 CM codes from the national database.

**Methods:**

We conducted a retrospective cohort study using the publicly accessible National Inpatient Sample (NIS) database from October 2015 to December 2017. Adult patients ( >/=18), who developed EB were included in the study. SAS 9.4 was used for univariate and multivariate logistic regression analyses.

**Results:**

There were a total of 75,465 patients included in the study who developed EB, among them 5440 (7.21%) patients with IE. In this cohort, 3,620 were males and 1,820 females, among them 3,955 (68.2%) were Caucasians, 620 (11.7%) were African Americans, 6,445 (8.80%) were Hispanic, 100 (1.9%) were Asian and 285 (5.4 %) were native Americans with IE. The majority of them were suffering from congestive heart failure (39.0%), valvular disease (22.0%), peripheral vascular disease (14.8%), chronic lung disease (16%) neurological complications (9.2%), diabetes mellitus with complications (13%) renal failure (32%), obesity (11.0%), and/or drug abuse (8%). In-hospital mortality was 13.7% (p< 0.0001), which was most likely due to the underlying co-morbidities, with an average length of stay of 15.8 (7-18) days and an economic burden of 48,850.3 (16,104–54,410) USD in comparison to patients without IE, who had in-hospital mortality 10.9%, average length of stay 13.6 (5-15) days, and economic burden 49,638 (11009-36665) USD. Age-adjusted mortality among the patients with Infective Endocarditis was 1.2 (1.07-1.28).

**Conclusion:**

Based on the results from our study, we found that patients with enterococcus bacteremia in appropriate settings should be screened for infective endocarditis to decrease the in-hospital mortality, length of stay, and economic burden.

**Disclosures:**

**All Authors**: No reported disclosures.

